# Epithelial Ovarian Cancer: Focus on Targeted Therapy

**DOI:** 10.1155/2010/171425

**Published:** 2011-07-05

**Authors:** M. Markman, Jalid Sehouli, Charles F. Levenback, Dennis S. Chi

**Affiliations:** ^1^The University of Texas MD, Anderson Cancer Center, Houston, TX 77030, USA; ^2^Frauenklinik der Charité, Universitätsmedizin Berlin, 13353 Berlin, Germany; ^3^Memorial Sloan-Kettering Cancer Center, New York, NY 10065, USA

Over the past five decades ovarian cancer has been of considerable interest to clinical cancer investigators due to the fact that it is among the most chemosensitive of all solid tumors [1]. Unfortunately, despite the advances in the chemotherapeutic management of this malignancy, the large majority of patients ultimately recur, progress, and ultimately die as a direct result of complications of the disease process. Thus, there is a critical need to find novel agents that may favorably impact the natural history of ovarian cancer. 

 In recent years there has been a particular focus in the cancer research community to discover and subsequently develop clinically active drugs that are capable of specifically *targeting* biological pathways relevant in a particular tumor type (e.g., ovarian cancer) and even within a specific patient with that type of cancer (so-called, “personalized medicine”). Research in this arena in ovarian cancer remains in its early stages although a number of quite exciting developments have recently been reported in the peer-reviewed medical literature that suggest the realistic potential that this novel general class of drugs will soon become important components of “standard-of-care” in the management of this difficult malignancy.

 In this special issue, investigators from around the world have contributed to this literature by summarizing a number of important developments. In the papers of this special issue, an initial discussion of the role of surgical cytoreduction in the malignancy is followed by an overview of the management of recurrent ovarian cancer and the relevance of molecular abnormalities in specific ovarian cancer subtypes.

 This is followed by several excellent and comprehensive overviews of the possible roles of targeted therapy in ovarian cancer, the potential impact of antiangiogenic drugs, epidermal growth factor and PARP inhibitors, disruption of insulin and glucose pathways, and novel treatments affecting histone deacetylase and metastatic colonization, as well as an innovative approach to immunotherapy in the malignancy.

 The peer-reviewed papers in this special issue provide important insight into both the current and future management of epithelial ovarian cancer.

*M.Markman* *M.Markman* *Jalid Sehouli* *Jalid Sehouli* *Charles F. Levenback* *Charles F. Levenback* *Dennis S. Chi* *Dennis S. Chi*
